# The Role of Service Recovery in Post-purchase Consumer Behavior During COVID-19: A Malaysian Perspective

**DOI:** 10.3389/fpsyg.2021.786603

**Published:** 2022-02-02

**Authors:** Muhammad Mazhar, Ding Hooi Ting, Ali Hussain, Muhammad Aamir Nadeem, Muhammad Asghar Ali, Umaima Tariq

**Affiliations:** ^1^Department of Management and Humanities, Universiti Teknologi PETRONAS, Seri Iskandar, Malaysia; ^2^School of Management, Universiti Sains Malaysia, Gelugor, Malaysia; ^3^Ziauddin University, Karachi, Pakistan

**Keywords:** COVID-19, service failure, service recovery, repurchase intention, switching intention, customer assistive intent

## Abstract

The purpose of this study is to investigate the incidence of service failure in rendering service process during COVID-19. It further explores the outcomes of service recovery offered to customers in case of service failure. Like other businesses, webstores have also faced the challenges in their efforts to satisfy their customers during COVID-19. Service failure has increased due to unexpected circumstances produced by this pandemic. It has become necessary for the webstores to retain their dissatisfied customers by reconsidering their service strategies. Relevant data for the purpose of this study were collected through questionnaires from 383 respondents by using online channels. The online channels were exclusively employed for maintaining the safety of respondents during COVID-19. Respondents for this study were online shoppers who encountered service failure during COVID-19. The results indicated that the incidence of service failure has increased due to an increase in online shopping during COVID-19. Some customers tend to repurchase from the same webstore. On the other hand, some customers do not want to purchase again from the same seller and decided to switch to the alternative webstore. Based on the findings, new strategy for online shopping service providers was introduced. This strategy will be helpful for the online service providers to increase their profitability by retaining their dissatisfied customers. Service providers can minimize the number of customers switching to other webstores by reducing the events of service failure. Customer’s assistive intent can also be helpful for service providers to increase the efficiency of service recovery. Conducting a proper follow-up after providing service recovery can also reduce the switching of customer. It will be helpful for service providers to understand the customers’ expectations before recovery process and their feeling after getting service recovery.

## Introduction

With the fear of COVID-19, coupled with the restricted movement orders, the condition has forced people to move toward online shopping rather than physical shopping (Liu and Lin, 2020). Customers’ shift from offline to online has resulted in a heavy load of orders on webstores. Some webstores were not prepared for such an unexpected order hike. Such webstores faced the challenge of coming up with strategies for providing satisfactory services to a swelling number of customers. This heavy traffic of customers, delays in transportation due to COVID-19, and unavailability of working staff might also have caused service failure in fulfilling orders (Shamim et al., 2021): for instance, delivery failure (delivery later than promised, wrong item delivered, or damaged items delivered), system failure (navigational problem, insufficient product information), product quality failure (poor product quality), website security failure (credit card fraud, sharing personal information to e-retailers), payment problems (payment overcharged, confusing purchasing process), and customer support failure (poor communication, unfair return policies) (Holloway et al., 2005).

Previous research linked with service recovery and customers’ response did not show the clear picture of customer response especially under special circumstances like COVID-19. The relevant literature suggests that service recovery (compensation or apology or both) can remove the effects of service failure (Migacz et al., 2017). However, this assumption does not work in all contexts and all situations of service failure. Customers always have concerns regarding service delivery, quality, and privacy of personal information during online shopping (Tsai and Yeh, 2010). Also, the service recovery strategies used in traditional market are not applicable in e-commerce (webstore) service industry (Luo et al., 2017). In physical stores, more interaction between seller and customer is an opportunity for seller to satisfy their customer by explaining and offering best service recovery (Javed et al., 2020). Also, customers are in a better position to immediately share their concerns regarding service failure. In online shopping, less interaction creates problems for both seller and customers. Due to time and space constraints, service providers have to pay more attention of customers’ psychological expectations regarding service recovery (Luo et al., 2017). Unfortunately, until now, same service strategies are implemented in online and offline businesses. However, service providers face difficulty in retaining their dissatisfied customers in online services by implementing the same strategies that are employed offline. The reason is that the service providers offer service recovery only to the complainers and do not get feedback from all customers who might face service failure at any stage of rendering service but did not complaint to service provider. Also, occasionally, customers do not satisfy with offered service recovery. The current study contributes to the existing literature by spotting the service failure at different steps of service process. Furthermore, the current study adds to the existing knowledge by introducing new service recovery strategies.

In case of service failure, usually customers complain to the service providers. Webstores practice service recovery as a tool to redress the service failures and retain their existing customers (Almarashdeh et al., 2019). However, the availability of multiple webstores (competitive environments) has created a challenging environment for webstores to retain their customer (Calvo-Porral and Lévy-Mangín, 2018). Customers can easily move to other webstores in a single click (Elgendy et al., 2019). Service recovery might be beneficial for customers, but to avoid future inconvenience, the customers may still switch to other webstores (Li C.J. et al., 2020). Further, some of customers do not bother to complain to the webstores because of complex and time-taking process (Istanbulluoglu et al., 2017; Li C.J. et al., 2020). So, in both cases, complaining and non-complaining customers may switch to alternatives even after getting service recovery. The question that arises here is, “how to retain customers when there is service failure?” “How to ensure that customers will remain loyal to the webstore amid competitions?”

Customer’s retention and loyalty are necessary for survival of companies (Al-Ghraibah, 2020). To retain existing customers is less expensive rather than attracting new customers. There is no universal formula for retaining existing customers (Al-Ghraibah, 2020). There might be different factors (dissatisfied with service recovery, low or no switching cost, available alternatives) that can influence the customers’ switching behavior (Liu et al., 2016; Sakunia and Parikh, 2020). The webstores have already existed for decades, but currently do not have any clear customer retention strategy. Researchers suggested that service recovery impacts on customer satisfaction, loyalty, and future intentions (Du et al., 2010; Komunda and Osarenkhoe, 2012; Osarenkhoe and Komunda, 2013) but would this be applicable to the context of webstores that are substitutable? Studies reveal that even excellent service recovery is not enough to restore attitude and behavior of customer (Lee and Zahn, 2014). The varying results of service recovery and contradictory opinions on the matter suggest improvements in the service recovery strategies. The question remains that how would this be relevant to the webstore context.

Service recovery is not the solution of service failure in every context. Customers might not repurchase the services even after attaining the service recovery (Lee and Zahn, 2014). Therefore, there is a need to alter the strategies for retaining customers. To overcome this problem, the current study focuses on the occurrence of service failure at different stages during the delivery of service. In this study, we focused on the complete service rendering process starting from the order till using the service/product. Furthermore, the current study examines which service recovery strategies are implemented by service providers to overcome service failure. This study introduces customer’s assistive intent as a new strategy to overcome the service failure in particular situation. Webstores must be understanding the customers’ expectations regarding service recovery to retain them (Hussain et al., 2020). A survey was conducted from dissatisfied online shoppers to understand the service recovery strategies. New strategies of service recovery were introduced based on the findings.

## Literature Review

The aim of this study is to investigate the customer’s future purchasing intentions when encountered with service failure during COVID-19. In normal conditions, online shopping is different than in COVID situation. Normally, customers have more options to purchase offline and online. Even in online, they have much time to wait for receiving their service/product. In COVID situation, customers have to rely only on webstores. Normally, customers can go to malls and enjoy the environment, and they can check products physically. However, in COVID situation, customers are afraid of pandemic and they prefer to purchase online. In online shopping, there are multiple issues that a customer might face. So these factors might become the cause of service failure that further might result in complaining or exit behavior. In this study, we tried to understand the factors behind customers’ decision and how these factors affect the purchasing decision of customers? This section consists of current research literature regarding the effect of service failure, service recovery process, customer satisfaction, and future behavioral intentions.

### Service Failure in Online Shopping

COVID-19 outbreak forced the customers to purchase necessities online rather than offline. Malaysia announced its first case of Corona on January 25, 2020. After a rapid increase in Coronavirus cases in March, the government imposed movement control order (MCO) on March 18, 2020 (Isa et al., 2020). For minimizing the chance of spreading coronavirus, government ordered many businesses to be closed (Martin-Neuninger and Ruby, 2020). Only specific businesses like grocery stores can remain open so that minimum customers come out for purchasing their necessities. During the MCO, many businesses were closed. During that time, there was a drastic switch to webstores. E-commerce is considered as an essential service in Malaysia (Isa et al., 2020). Pandemic altered all the traditional shopping behaviors (Hasanat et al., 2020).

During the MCO, online shopping became popular medium of purchasing not only for customers but also for traders (Isa et al., 2020). Social distancing also forced customer to purchase online so that they can save their time (Hasanat et al., 2020; Isa et al., 2020). During MCO, different webstores like happy fresh, Lazada, and Shopee experienced increase in orders. A 10–15% increase in orders has been reported by webstores, which created troubles for suppliers to store and supply demanded products (Mymetro, 2020). As online shopping percentage increases, service failures are also increased in different ways. For example, required products were out of stock, ordered items were delivered late, wrong items were delivered, damaged items were delivered, and there were website connectivity issues, online payment transaction issues, and personal information privacy issues. Statistics shows that there were 11.9 million webstore users in 2019. The number of users has been increased to 14.4 million until now. The revenue for online shopping has been increased by 89% in 2020. Lazada had the highest number of active users in the third quarter of 2019. Like other companies, Lazada is also contributing in economy of Malaysia to sustain in COVID-19 (Müller, 2020). For example, Lazada also helped in collecting donations for homeless people. As well as offered SMEs to use Lazada’s platform for selling their products. Lazada is a big online shopping webstore and has a majority of active online customers. However, Lazada itself was not well equipped to cope with the current situation of COVID-19. Therefore, Lazada also faced different problems in delivering satisfying services to its customers, such as late in the delivery of orders. Therefore, Lazada was chosen for the current study.

Many researchers have contributed to consumer behavior during COVID-19. They discussed the problems that are faced by the customers while using online shopping during COVID-19. For instance, customers faced trouble in getting fresh products and vegetables (Li J. et al., 2020), consumers’ grocery purchasing behavior during COVID-19 (Grashuis et al., 2020; Hall et al., 2020; Li J. et al., 2020), spending pattern (Andersen et al., 2020), food purchasing habits (Richards and Rickard, 2020), and online shopping behavior (Hasanat et al., 2020; Kim, 2020). In the era of pandemic, while customers are already facing troubles to get their required products, issue of service failure ruins the customer’s relationship with service provider. Companies must consider the issue of service failure during COVID and think differently to retain their customers. The following table provides an overview of research conducted on consumer behavior during COVID-19. In recent studies conducted during COVID-19, researchers mainly focused on purchasing trends of customers, shifting from offline shopping to online shopping, online grocery shopping, and technology adoption. However, the concept of service failure and service recovery is oversighted.

Service failure occurs when the customers are dissatisfied with service/product or its delivery process (Maxham, 2001). When a customer received a wrong service, it goes and leaves customers feeling negatively about the service experience; as a result, a service failure has happened (Gelbrich and Roschk, 2011). Service failures increase the switching intention of customers as well as decrease the loyalty toward service provider (Pieters et al., 2019). When a customer faces a service failure during his purchase, their emotions hurt, and the customer will try to avoid purchase due to the fear of the repetition of service failure (Kamble and Walvekar, 2019). The customer induced to seek services from other available service providers. The negative response of service providers toward the service failure produces negative outcomes, such as negative WOM and decrease in profit (Tax et al., 1998; Bitner et al., 2000; Tronvoll, 2011), and spreads in market like a virus (Zhu et al., 2021). If service provider cannot redress the dissatisfied customer properly, he will not only switch the services of service provider but also share his bad experience with his social circle (Cai and Qu, 2018). Service failures are inevitable because of the integral inconsistency of service performance (Zeithaml et al., 1990). In response to the service failure, the customers might complain to the service provider. Poorly handled complaints are certainly not forgotten, and these customers are vulnerable to defection (Rotte et al., 2006). Therefore, service providers need to handle the complaining behavior properly to retain their customers and ultimately maximize their profit. A defensive strategy to keep the existing customer is less expensive compared to the offensive strategy that attracts new customers. Attracting new customers is five times more expensive as keeping an existing one (Timm, 2001). It has been observed that if a company brings 5% of its angry customers, then the profit will boost from 25 to 95% (Kotler, 2003; Manzano-Machob, 2013).

A number of existing studies have investigated service failure, including delivery failure (delivery later than promised, wrong item delivered or damaged items delivered), system failure (navigational problem, insufficient product information), product quality failure (poor product quality), website security failure (credit card fraud, sharing personal information to e-retailers), payment problems (payment overcharged, confusing purchasing process), and customer support failure (poor communication, unfair return policies) (Holloway et al., 2005; Holloway and Beatty, 2008).

Though, different studies reported different types of service failure in their findings. The service failure at any stage of complete service obtaining process, starting from ordering till consumption, is overlooked. In this study, we have tried to consider the complete service-acquiring process and the response of customers at different stages of this process. Based on the above discussion, the following hypothesis is proposed:

H1: Service failure positively influences the complaining behavior.

### Customers’ Complaining Behavior

Complaining can be behavioral or non-behavioral (Singh, 1988). In behavioral study, the customer complain to company (seller, retailer, website, or service provider), third-party (legal and consumer protection organizations), or friends and family (Singh, 1988). On the other hand, customers face service failure but do not launch a visible complaint, which is non-behavioral complaining response. When customers have encountered a service failure, they want redress to vent their frustration and anger. Now in the pandemic situation, companies are still focusing on the same strategies of service recovery (compensation, explanation, and apology). But customers’ behaviors and expectations are changed due to current situation. It is very hard to encourage customers to complaint to service provider as they consider it more difficult in online setting. Comparatively in offline store, the customer can elaborate his/her complaint in detail. In online complaints, more psychological effort is required as compared to offline complaints. Customers have to wait for long to get resolution of their complaints. In offline setting, customers can go to service provider and can confirm the status of their complaint, but in online setting, 50% of complaints are ignored by the sellers (Rosenmayer et al., 2018).

In online complaining, non-verbal communication and face-to-face interaction are not available, which have a psychological impact on the customer (Rosenmayer et al., 2018). Criticality in launching a complaint is a big issue in complaining to webstore. In the online complaining system, many customers do not know how to launch and follow up a complaint (Järvenpää, 2017). Another factor in non-complaining behavior is an alternative to same services that are available in market. Customers silently switched to other webstores and do not bother to the complaint. Customers who complaint to webstores do not get proper response from the webstores that might be due to less staff available to respond to customers timely. Customers complained to the webstores, but they did not get service recovery as per their expectations. The above-discussed issues might be the factors, which leads the customers to switch the webstore to fulfill their needs.

Complaints are the opportunities for the companies to improve their services or redesign their modes of providing services (Turner, 2018). Complaining helps in many ways to service providers. If customer does not complain to the service provider, it causes loss not only for the customers but also for the service provider (Sands et al., 2020). In previous studies, it is mentioned that after service failure, customers complain publicly or privately (Istanbulluoglu et al., 2017). It is still needed to study the complaining behavior of customers in online shopping in special circumstances like COVID-19. In normal situation, the customer can purchase from offline store to fulfill their needs temporarily until they acquire service/product from webstore. In COVID situation, customers are more careful about the health of their families and community so they prefer to purchase online. Though it is easy to purchase through webstores, it also has its own shortcomings. If customers do not get proper response from the webstore, they do not bother to complain again they exit silently. Customers have more choices in current competitive era (Liu and Atuahene-Gima, 2018). So the complaining method must be very easy so that a common. Customer who does not have much grip on technology cannot launch complaint easily. Sometimes customers only do not complaint due to the lengthy procedure of launching a complaint (Järvenpää, 2017). COVID-19 has forced the companies to decrease their employees to minimize the risk of pandemic (Blustein et al., 2020). Further, the government has implemented the lockdown and restricted the movement of people (Isa et al., 2020). These restrictions have created problems in transportation. On the contrary, the increase in orders has created a severe problem for companies to fulfill the customer’s requirement on time. The fear of COVID-19 has forced the people to order consumer products through online channels (Grashuis et al., 2020). If these items were not delivered on time, the customer found the alternatives and switched to other service providers. The complaining behavior of customer provides a chance to webstore to make their services better and retain their customers by providing a better service recovery. Webstores encourage customers to report their complaints to webstores so that they can make error-free services. Complaining behavior has a direct link with service recovery; hence, the following hypothesis is proposed:

H2: Complaining behavior has a positive effect on service recovery.

### Service Recovery in Online Shopping

In a highly competitive service environment, it has become very difficult for organizations to attract their dis-satisfied customers and develop an effective strategy for providing service recovery to retain their customers (Migacz et al., 2017). In a competitive environment, customers have more power to select alternative product/service. Previous studies show that an interest in service recovery has increased because service failure experience often leads to customer switching. Although the first rule for providing services should be to do things in their right manners, Zeithaml et al. (2006) have developed different strategies for satisfying customers to help the marketers: act quickly, encourage and track complaints, treat customers fairly, cultivate relations with customers, and provide explanation.

When service failure occurs, it becomes essential for webstore to reacquire dissatisfied customers so that financial and reputational losses can be minimized. Providing service recovery to dissatisfied/complaining customers is an opportunity for retailers to model the perception of customers about their brand (Turner, 2018). In the literature, it is revealed that poor service recovery was offered to the customers. For example, some online retailers respond only to a half of the complaints they received, and in response to those complaints, they just offered apology or empathy (Rosenmayer et al., 2018). Webstores must be careful about the complaints of customers as well as their expectation regarding service recovery. The following table provides a review on studies of service recovery in online shopping context. If a customer is dissatisfied with service failure, she/he might be dissatisfied/satisfied with service recovery (Tarofder et al., 2016). 50% of complains are dissatisfied with the service recovery provided by service provider (Bradley and Sparks, 2012). It is also reported in a study that only 30% customers are recovered by the service provider after service failure (Michel and Meuter, 2008). A customer’s future intention is based on type of service failure and recovery strategy (Riscinto Kozub et al., 2014). It is very important for service providers to understand the expectations of customers regarding service recovery (Zhu et al., 2021). In another study, it is reported that 70% of service recovery efforts were wasted due to misunderstanding the expectations of customers (Maher and Sobh, 2014). Previous studies investigated the issue of service failure and repurchase behavior. They posit that after a service failure, the customer tends to spread NWOM and have less intention to repurchase (Petitjean, 2013). It is not necessary that in all cases, the customer will continue purchasing from the same service provider in the future (Harrison-Walker, 2012).

The service recovery strategies are not same for online customers and offline customers (Baron et al., 2005). The environment of online/offline shopping plays an important role in providing service recovery (Baron et al., 2005). If choice of recovery were provided to customers, it would have a significant impact on overall satisfaction with webstore (Chang, 2008).

### Service Recovery and Switching Intentions

Switching is defined as “replacing or exchanging the current service provider with another service provider” (Bansal and Taylor, 1999). Switching is considered as opposite to loyalty (Singh and Rosengren, 2020). Loyalty focuses on positive outcome of purchase experience with webstore, and a loyal customer must purchase from the same seller in the future. Switching behavior shows the negative outcome of customer that leads customer to leave. Technological advancements changed the business trend nowadays. Companies are shifting their businesses toward online channels to achieve competitive advantage in the market. Currently, customers have many options to fulfill their necessities but switching behavior is harmful action (Istanbulluoglu et al., 2017). In the literature, it is argued that attracting new customers is five to eight times more expensive than retaining new customers (Wu and Huang, 2015). Service failure is one of the major factors that affects switching intentions (Pieters et al., 2019). Literature is evident that after service failure if service provider offers service recovery to the customers, it will remove dissatisfaction and make the customer loyal, but due to low switching cost, customers shift toward other webstores with a single click (Zhu et al., 2021).

In online shopping, less or no human interaction creates difficulties for customers to express their problems to the service provider, which leads to high switching behavior (Holloway and Beatty, 2003; Javed et al., 2020). In online shopping, if service provider provides service recovery to the customer but the customer was expecting more than offered, in this situation the customer will switch even after getting service recovery (Maher and Sobh, 2014). Effective service recovery provided by webstores can reduce the switching rate of customers. It will be only possible if webstores keep in touch continuously during whole process of taking service. Timely and continuous response to customer queries might reduce the switching intention. Based on the discussion, we proposed the following hypothesis:

H3: Service recovery positively affects the switching intention.

### Service Recovery and Repurchase Intention

Profit maximizing is the main objective of all organizations. To attain this objective, they try to make their customer loyal and retain them. Currently, researchers are focusing more on customers’ anti-consumption behaviors (Curina et al., 2020). Anti-consumption behavior explains the impact of negative emotions evoked by service failure and their influence on loyalty, repurchase intention, and frequency of use (Jayasimha et al., 2017; Zarantonello et al., 2018). Risk of service failure also affects the repurchase behavior of customer (Lăzăroiu et al., 2020). From a managerial perspective, it is necessary for service providers to deal with customers’ service failure issues effectively. It directly or indirectly affects customer’s repurchase intention. In traditional markets, different service recovery strategies are using to improve customers’ repurchase intention. However, in online shopping, customer’s behavior toward webstores is different. If customers did not get service recovery as per his/her expectations, he will not repurchase from that webstore.

Currently, due to the COVID-19, customers are remaining at their homes and they have more time to search their required products available on different shopping sites. So, in this situation, when a customer has faced service failure, there is higher chance of his switching. For example, if a customer purchases from an online shopping during a promotion campaign but the received product is not as per his expectations, he contacts customer care and asks for compensation. The service provider promises a refund. When the customer gets the compensation, he is likely to revisit the same webstore for repurchase. However, once the promotion campaign has ended, he observes that the same product is available at a higher price. In that case, he will not repurchase and switch to other available options. Repurchase intention is directly linked with efficient service recovery. In current situation of COVID, webstores might focus on some additional strategies so that customer’s repurchase intention can be enhanced. Based on the above discussion, the following hypothesis is proposed:

H4: Service recovery positively influences the repurchase intention.

### Expectation–Disconfirmation Paradigm

Customers always try to get the maximum value of service/product (Lu et al., 2020). They expect more from the service provider (Lu et al., 2020). If a customer gets as per his expectations, it makes him satisfied, which in turn leads to future purchase, and *vice versa* (Michel and Meuter, 2008). The expectation–disconfirmation theory has been used widely in different contexts to investigate the customer’s post-purchase behavior (Ayanso et al., 2015). The expectancy confirmation theory (ECT) is developed to measure the satisfaction of customer and to check the impact of satisfaction on the willingness of customer to repurchase (Ayanso et al., 2015). As per expectancy disconfirmation theory presented by Oliver (1977), confirmation happens when performance of service/product matches the expectation. When a customer has a bad experience with a service/product, it causes negative disconfirmation, and positive disconfirmation happens when a customer gets better service/product performance than expected. Expectancy–disconfirmation model explains the comparison between expected and actual service/product performance, further resulting in dissatisfaction/satisfaction (Oliver and DeSarbo, 1988). Customers make the evaluation of actual performance of service/product on the basis of his/her expectations and results could be any one of the following three outcomes: (1) Positive disconfirmation (if actual performance of service/product is higher than expected), (2) confirmation (if actual performance of service/product is equal to expectations), and (3) negative disconfirmation (if actual performance of service/product is lower than expected). Based on the three outcomes, the customer further decides its future intentions. If the customer is highly satisfied, he becomes loyal customer, and as the result of dissatisfaction, the customer will switch the service provider. Many scholars have identified that expectancy–disconfirmation theory has an effect on customer’s satisfaction (James et al., 2015; Sarkar et al., 2015), which leads to repurchase or switching behavior. This paper studies the probable future intentions of customers after facing service failure. Customers complain to service provider or their friend and family to vent their frustration. Service provider will provide service recovery to customers who faced service failure. So, if the service recovery will be as per expectations of customer, it will be the confirmation stage. In this situation, a customer might repurchase or switch. If the customer receives less service recovery with respect to his expectations, it will be the negative disconfirmation. In this situation, more chances are toward switching webstores. More than expected, service recovery is positively disconfirmed. Positive disconfirmation might lead to repurchase intention. A customer decides his future purchase from the same seller or switching to other webstores based on the evaluation of the expected and actual service recovery.

### Research Methods

A questionnaire was constructed to collect data from the targeted customers. The questionnaire consisted of multiple items based on the previous literature and was divided into two parts. The first part of the questionnaire was about the basic information of respondents. Basic information like gender, education, occupation, and age was gathered to understand the characteristics of respondents. All questions related to basic information formalized on a nominal scale were used to measure the respondents’ characteristics. The second part of questionnaire includes the questions related to variables of the research. For measuring the customers’ response, a 5-point Likert scale was employed ranging from 1 (strongly agree) to 5 (strongly disagree). Items of different variables, service failure (Li et al., 2016; Das et al., 2019), complaining behavior (Singh, 1988), service recovery (Parasuraman et al., 2005), repurchase intention (Jeon et al., 2017), and switching intention (Nikbin et al., 2012) were adapted from the literature to compose the questionnaire.

All customers who purchased services or products through online shopping and faced any kind of service failure are the respondents of our study. Because we do not have the exact customers’ list who might be our respondents, we used snowball sampling technique, which includes purposive sampling. For data collection, snowball sampling is widely used by researchers (Browne, 2005; Baltar and Brunet, 2012), especially in those cases when we want to target as maximum as possible respondents with same characteristics, and it seems hard to reach (Sadler et al., 2010). The questionnaire was created on google forms (Sekaran and Bougie, 2019) and distributed in target respondents through using various online channels like WhatsApp groups, Facebook groups, and emails. It was crucial for us to collect data through online channels because limited opportunities for physical data collection were available during pandemic and lockdown. Collecting data using online channels allows us to ensure the safety of respondents as well as timely data collection.

### Data Analysis

#### Respondents’ Characteristics

A dataset of 383 valid responses was extracted from 407 received responses in initial screening for empirical analysis. Twenty-four responses were excluded due to inefficient responding (Dunn et al., 2018). The majority of respondents (64.8%) were male, aged 21–30 years (51.4%), Malay (82.2%), and a Master education level (36.0%). The frequency distribution of respondent’s characteristics is presented in Table 3.

**TABLE 1:** Studies on online shopping during COVID-19.

Authors	Purpose of the study	Context of the study	Findings
Kim, 2020	Examines the effect of pandemic on the structural change in consumer behavior and digital transformation	Digital transformation in marketplace	Significant growth observed in E-commerce adoption during COVID-19
Li J. et al., 2020	Consumer behavior in food purchasing during the early stage of COVID-19	Customers’ grocery shopping behavior during COVID-19	Disturbance in food retailing is noticed during COVID-19
Hall et al., 2020	Evaluation of consumption displacement when consumer experience changes in availability of goods.	Grocery shopping behavior of customers in COVID-19	Storing behavior in COVID-19
Andersen et al., 2020	Investigate the spending and saving behavior during pandemic	Spending patterns of customers during COVID-19	Saving pattern is observed for future insecurities
Hasanat et al., 2020	Determine the impact of online business in Malaysia	Effects of COVID-19 on Malaysian’s online business	Online businesses faced trouble during COVID-19
Sheth, 2020	Impact of COVID-19 on the new and old habits of purchasing	New norms and standards for customers during and after COVID-19	Online shopping is the focus for purchasing
Richards and Rickard, 2020	Examine the nature of change in demand and supply of vegetables during COVID-19	Food purchasing habits *via* online channels.	Shifting of offline business toward online business
Grashuis et al., 2020	Investigate the grocery shopping behavior during COVID-19	Consumers’ grocery shopping behavior during COVID-19	Consumers are more preferring to buy online during COVID-19
Laato et al., 2020	To capture the unusual purchasing behavior during COVID-19	Customer purchasing behavior during COVID-19	Self-isolation and overload of online information lead to unusual purchase

**TABLE 2:** Service recovery studies on online shopping.

Authors	Purpose of study	Context of study	Data Collection Technique	Findings
Holloway and Beatty, 2003	To provide typology of service failure in online shopping and satisfaction level of customers after service recovery	Online retailing	Interviews and survey	Categorized service failure in online shopping in six groups (study 1). 54% customers complained and 25.6% customers planned to return online company (Study 2).
Baron et al., 2005	Focusing on e-commerce service failure and service recovery employed by service firm	Shopping websites	Survey	Grouped service failures in two groups and 10 categories. Most common error was packaging, and mostly customers were dissatisfied with size variation.
Holloway et al., 2005	To investigate the moderating role of purchasing experience in online shopping	Online shopping	Survey	Remedy offered has greater impact on the customer who has less purchasing experience.
Holloway and Beatty, 2008	Customers’ satisfiers and dissatisfiers	Online retailing	Survey	Four dimensions were suggested for dissatisfaction/satisfaction in online shopping, namely, customer services, fulfilment/reliability, website design/interaction, and security/privacy.
Chang, 2008	To find out the service recovery strategy for controlling customer satisfaction	Online bookstore	Survey	By providing choice of service, recovery can control the satisfaction of customer.
Kuo et al., 2011	To group service failure and strategies and identify best service recovery strategy for each service failure	Online auction	Survey	Failure incidents were classified into three groups and 18 subcategories and 10 service recovery strategies derived for service failure.
Rosenmayer et al., 2018	Different service failure types and service recovery strategies	Omni channel retailing	Document review of Facebook customer complaint and service recoveries	Customer complaints were triggered by varying service failure. Four dimensions appear valid for service recovery on Facebook.

**TABLE 3:** Respondent’s characteristics.

Criteria	Description	Frequency	Percentage (%)
Gender	Male	248	64.8
	Female	135	35.2
Age	Below 20 years	74	19.3
	21–30 years	197	51.4
	31–40 years	53	13.8
	41–50 years	31	8.1
	51–60 years	21	5.5
	Above 60 years	7	1.8
Highest education level	Certificate	33	8.6
	Diploma	55	14.4
	Bachelor	132	34.5
	Master	138	36.0
	Ph.D.	25	6.5
Nationality	Malay	315	82.2
	Other	68	17.8

### Common Method Variance

Common method variance usually occurs when data are collected from a single source in a single sitting (Yüksel, 2017). It may affect the structural relationships (Kline, 2015) and undermines validity (MacKenzie and Podsakoff, 2012). Two statistical controls designed were used to minimize the risk of CMV. First, Harman’s single factor method was implemented to detect the CMV in the data. The results indicate that 19.28% of the total variance by the single factor was the highest variance explained which is very less than the norm of 50% (Fuller et al., 2016). Second, the full multicollinearity test was done as per recommendation of Kock (2015). It reveals that pathological VIF values for all latent variables ranged from 1.000 to 2.850, which is well below the 3.3 threshold, validating that the data are free from CMV problem.

## Measurement Model Analysis

### Assessment of Reflective Constructs

Factor loading, Cronbach’s alpha, composite reliability (CR), average variance extracted (AVE), and discriminant validity were assessed to test the measurement model for reflective constructs. The findings are presented in Table 4. The values of factor loadings of each first-order reflective construct were higher than 0.5 for retaining the items (Hair et al., 2020). However, COMB2, COMB3, COMB5, INFF2, SYSF2, SYSF4, PRDF1, and PROSF1 were dropped due to low factor loadings. Cronbach alpha (α) > 0.70 and CR > 0.70 show a high degree of internal consistency, while AVE > 0.50 shows a high degree of convergent validity (Hair et al., 2019). Next to this, indicator multicollinearity was also evaluated through VIF test. The results tabulated in Table 4 reveal that VIF of each item is well below the limiting value of 5, suggesting that multicollinearity is not a problem in this study (Hair et al., 2020). Furthermore, heterotrait–monotrait ratio (HTMT) criteria were employed as per the recommendation of Hair et al. (2019) to determine the discriminant validity due to its superiority over other methods. Table 5 shows that HTMT values of each construct are less than the cutoff score of 0.90, indicating that all constructs are distinct. Hence, it can be concluded that all reflective constructs established a convergent and discriminant validity in this study.

**TABLE 4:** Measurement model.

Stage I: Results of the assessment of measurement model for first-order reflective constructs													
**First-order constructs**		**Code**	**FL**	**VIF**			**α**		**ρA**		**CR**		**AVE**

Complaining behavior		COMB1	0.670	1.818			0.824		0.838		0.871		0.531
		COMB4	0.642	1.463									
		COMB6	0.726	1.802									
		COMB7	0.824	2.735									
		COMB8	0.771	1.745									
		COMB9	0.726	1.509									
Compensation		COMP1	0.772	1.808			0.866		0.868		0.903		0.651
		COMP2	0.788	1.867									
		COMP3	0.779	1.835									
		COMP4	0.833	2.427									
		COMP5	0.859	2.631									
Contact		CONT1	0.827	2.188			0.885		0.887		0.916		0.686
		CONT2	0.774	1.889									
		CONT3	0.853	2.432									
		CONT4	0.832	2.241									
		CONT5	0.853	2.469									
Functional failure		FUNF1	0.744	1.847			0.844		0.845		0.885		0.562
		FUNF2	0.781	2.012									
		FUNF3	0.723	1.578									
		FUNF4	0.784	2.016									
		FUNF5	0.772	1.980									
		FUNF6	0.691	1.456									
Informational failure		INFF1	0.744	1.379			0.708		0.708		0.820		0.533
		INFF3	0.745	1.357									
		INFF4	0.714	1.288									
		INFF5	0.718	1.293									
Product failure		PRDF2	0.793	1.574			0.747		0.749		0.841		0.569
		PRDF3	0.729	1.362									
		PRDF4	0.779	1.494									
		PRDF5	0.715	1.319									
Process failure		PROF2	0.761	1.453			0.750		0.752		0.842		0.571
		PROF3	0.779	1.497									
		PROF4	0.762	1.419									
		PROF5	0.719	1.372									
Responsiveness		RESP1	0.834	2.078			0.836		0.836		0.890		0.670
		RESP2	0.818	1.985									
		RESP3	0.797	1.734									
		RESP4	0.824	1.823									
Repurchase intention		RPUI1	0.853	2.138			0.889		0.905		0.922		0.748
		RPUI2	0.883	2.320									
		RPUI3	0.857	2.492									
		RPUI4	0.866	2.474									
Switching intention		SWTI1	0.703	1.917			0.901		0.919		0.920		0.623
		SWTI2	0.819	2.412									
		SWTI3	0.833	2.313									
		SWTI4	0.752	1.731									
		SWTI5	0.776	2.297									
		SWTI6	0.806	2.307									
		SWTI7	0.829	2.589									
System failure		SYSF1	0.803	1.633			0.748		0.789		0.852		0.657
		SYSF3	0.779	1.587									
		SYSF5	0.848	1.366								

**Stage II: Results of the assessment of measurement model after generating second-order formative constructs**													

**Second-order construct**	**Relationship**				**VIF**	**Weight**		**Mean**	**S.D**	***t*-value**		***p*-value**	

Service failure	Functional Failure - > Service Failure				2.085	0.392		0.367	0.191	2.052		0.020	
	Informational Failure - > Service Failure				1.716	0.313		0.310	0.169	1.857		0.032	
	Process Failure - > Service Failure				1.834	0.334		0.325	0.177	1.886		0.030	
	Product Failure - > Service Failure				1.898	0.309		0.312	0.165	1.873		0.031	
	System Failure - > Service Failure				1.040	0.588		0.561	0.132	4.475		0.000	
Service recovery	Compensation - > Service Recovery				3.097	0.186		0.182	0.029	6.466		0.000	
	Contact - > Service Recovery				2.836	0.591		0.580	0.243	2.435		0.008	
	Responsiveness - > Service Recovery				2.259	0.318		0.316	0.180	1.762		0.039	

*FL, factor loading; VIF, variance inflation factor; α, cronbach’s alpha; ρA, dijkstra constant; CR, composite reliability; AVE, average variance extracted.*

**TABLE 5:** Discriminant validity (HTMT criteria).

	Compensation	Complaining behavior	Contact	Functional failure	Informational failure	Process failure	Product failure	Repurchase intention	Responsi veness	Switching intention	System failure
Compensation											
Complaining Behavior	0.290										
Contact	0.893	0.305									
Functional Failure	0.321	0.306	0.185								
Informational Failure	0.233	0.166	0.131	0.708							
Process Failure	0.264	0.263	0.116	0.712	0.797						
Product Failure	0.314	0.319	0.178	0.818	0.663	0.714					
Repurchase Intention	0.309	0.074	0.245	0.066	0.125	0.078	0.067				
Responsiveness	0.843	0.342	0.798	0.162	0.123	0.067	0.150	0.221			
Switching Intention	0.234	0.614	0.332	0.308	0.092	0.165	0.262	0.049	0.258		
System failure	0.221	0.320	0.209	0.204	0.171	0.118	0.213	0.057	0.254	0.188	

*Threshold value 0.90.*

### Assessment of Formative Constructs

This study proposed service failure and service recovery as a type two higher-order (reflective-formative) constructs. Therefore, a disjoint two-stage approach, as suggested by Shmueli et al. (2019), was adopted, which was employed in three steps. In first step, convergent validity was checked by means of redundancy analysis. The findings show that service failure and service recovery have a correlation of 0.792 and 0.828 with its global item, respectively, which is sufficiently above 0.70. It indicates convergent validity established for higher-order (reflective-formative) constructs. In the second step, the multicollinearity of the indicators (VIF) was used to determine the formative measure. The results are tabulated in Table 4. The VIF value is well below the cutoff value of three for all measures (Hair et al., 2019), meaning that collinearity is not a serious concern in this study. In the last, a bootstrapping procedure with 5,000 subsamples was used to assess the significance of weights. The results in Table 4 indicate that the weights of all indicators of both higher-order reflective formative constructs were significant. As such, it can be concluded that the measurement model was validated.

### Structural Model Analysis

Following the measurement model, structural model was assessed to analyze the statistical significance of path coefficients, explanatory power, predictive relevance, and their effect sizes. A bootstrapping with 5,000 subsamples was carried out to test significance of proposed relationships. As shown in Table 6, all four hypothesized relationships are statistically significant. Service failure is positively related to complaining behavior (H1: β = 0.363, *p* = 0.000). Similarly, complaining behavior is positively related to service recovery (H2: β = 0.294, *p* = 0.000). Moreover, the relationships between service recovery and switching intention (H3: β = 0.300, *p* = 0.000) is positively significant. Besides, the effect of service recovery on repurchase intention (H4: β = 0.3245, *p* = 0.000) is also positively significant. However, this effect is less significant as compared to the effect of service recovery on switching intention. Furthermore, effect sizes (*f*^2^) are also tabulated in Table 6, which indicates the effect sizes of weak to medium range.

**TABLE 6:** Hypotheses testing.

Hypothesis	Relationship	Beta value	Mean	S.D	*t*-values	*p*-values	95%	95%	Decision	*f* ^2^
							CI LL	CI UL		
H1	Service Failure - > Complaining Behavior	0.363	0.376	0.047	7.731	0.000	0.257	0.424	Accepted	0.152
H2	Complaining Behavior - > Service Recovery	0.294	0.303	0.061	4.803	0.000	0.177	0.378	Accepted	0.095
H3	Service Recovery - > Switching Intention	0.300	0.307	0.056	5.400	0.000	0.191	0.381	Accepted	0.099
H4	Service Recovery - > Repurchase Intention	0.245	0.248	0.069	3.563	0.000	0.119	0.352	Accepted	0.064

*S.D, Standard deviation; CI, Confidence interval; LL, Lower limit; UL, Upper limit.*

Further, coefficient of determination (R2) was used to evaluate the explanatory power of dependent variables by the independent variables. The R2 values are given in Table 7, which show moderate to weak explanatory power of the model (Cohen, 2013). Similarly, blindfolding procedure was used to assess the predictive relevance of this study. Findings in Table 7 reveal that Q2 values are less than 0.25, which is an indicative of low predictive relevance in this study (Hair et al., 2019).

**TABLE 7:** Predictive relevance and coefficient of determination.

Variable	Coefficient of determination	Predictive relevance
	*R* ^2^	Q^2^
Complaining behavior	0.132	0.102
Service recovery	0.086	0.035
Switching intention	0.090	0.016
Repurchase intention	0.060	0.027

## Findings and Discussion

COVID-19 altered the consumer buying behavior globally (Ali, 2020). The companies shifted their businesses toward online channels. E-commerce was also affected by COVID-19 significantly, although online shopping increased in pandemic (Bhatti et al., 2020). The customers preferred online shopping during COVID-19. Online shopping is safer, cheaper, and more time-saving and fear of corona forced customers to buy daily routine life products through online shopping (Abiad et al., 2020). Though customers were already familiar with online shopping before the pandemic, in lockdown it became necessity of customers to buy online. Smartphones and Internet have made the online shopping easier for customers. They can place orders from anywhere and delivered at their desired address. The increase in online shopping also creates challenges for webstores (Hasanat et al., 2020). Customers faced many service failures during their purchase processes like website overloaded, stock out of order, and extended delivery time (Abiad et al., 2020).

While customers faced any kind of service failure during purchase cycle, it creates a negative effect in their minds. In prior studies, varying results were provided by the researchers regarding the effect of service recovery. In offline businesses and online businesses, the service recovery strategies are different. Traditional service recovery strategies cannot implement everywhere in all businesses. Similarly, in normal conditions and pandemic situation, the service recovery strategy should be different as per the severity of service failure and expectations of customers. The findings of our studies showed that customers who complained to the webstore got service recovery either in monetary or in non-monetary form. After getting service recovery, only few customers are willing to repurchase from the same webstore. A high number of customers switched to another webstore. Our findings revealed that though service recovery has a positive effect on customers’ post-purchase behavior, the majority of customers are not happy with the service or service recovery they got from the webstore.

The quality of product is also affected due to COVID-19. The possible reason might be the shortage of time and increase in orders. To provide a detailed informative video with all the products on the webstore can also help the customers to take purchase decision. Webstore should also encourage customers to share their experiences in a short video on their website, which will also be very helpful. The customers who received the wrong product complained to the webstore. They were dissatisfied from the service recovery strategy of webstore. In this situation of service recovery offered by webstore but customer is still dissatisfied. Though service provider compensates him by refunding his full amount, the customer also invested his precious time in whole process, and he was expecting more than this. Here, if service provider engages customer in service recovery process, this will help service provider to understand what customer’s expectations are. Customer’s assistive intent can help webstore to understand better service recovery for their customers.

**FIGURE 1:**
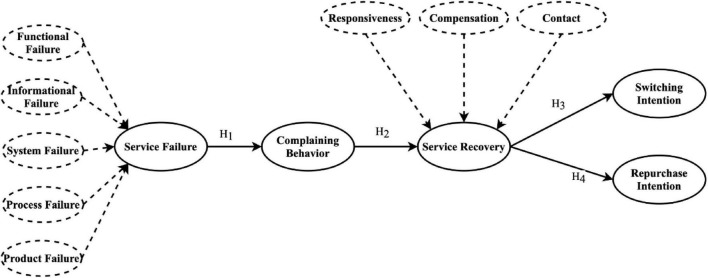
Theoretical research framework.

Millions of people have lost their jobs that make trouble for companies to fulfill customers demand on time (Jiang and Wen, 2020). Customers who have faced service failure contacted to the webstore. These customers are still dissatisfied from the response of the webstore representative. Customer support center is a department that can be run by maintaining social distance; even representative can do their work from home. If the customer receives a quick resolution response, it will make customer happy, and he might choose the same webstore for his future purchase. Webstore is a place where different sellers sale their products and customers have many options to choose the right product as per their requirements. It was noticed that a variety of products are available by different sellers. It is hard to judge the quality of service only by the name of their sellers. Reviews of the products are also helpful for the selection of product; however, negative reviews make the customers more conscious. Customers do not want to take risk of service failure, so they try to get best option. For this purpose, webstore must have a check on seller’s product quality so that the customer gets better services every time.

Maximum customers are purchasing from the online webstores due to COVID and MCO, so it makes the websites overloaded and customers faced problems in searching their required products. Sometimes webstore showed a product as available for sale but when the customer made a transaction to order that item, the system showed that the selected item is out of stock. It is also noticed that pictures of products were showing discount offers but in actual no offer was available. In such cases, webstore must increase the performance of their website so that the customer does not face such issue that leads them to switching to another webstore. Respondents suggest that webstores should enhance their IT-related capabilities and be vigilant for trouble shooting in case of any problem reported by customers.

The COVID-19 pandemic has created a fear among customers. According to reports, the germs of coronavirus are active on the surface for several hours (Goldman, 2020). Some of the customers were reluctant to order online because the product might be infected after delivery. The respondents suggested that the products should be disinfected before delivery. Second, the customers were also afraid to receive a product from the delivery person, as the delivery person might also be infected, and he/she does not know about that. Upon asking the suggestion, the respondents replied that the service providers should deliver the products through drones to avoid the pandemic. The webstores and customers both know that there are delays due to COVID-19. However, the approximate delay should be mentioned on the webstore. This will help the webstore to deliver order in stated time. The customers will also not be irritated because of unexpected delivery delay.

Customers’ assistive intent is an important variable that we suggest incorporating in service recovery strategy. It would have positive impacts on customer retention and reduce the switching behavior. It would be helpful to influence the perception of customer that will improve patronage intention toward webstore. In the context of service recovery, the customer’s assistive intent refers to the “customers help webstore by incorporating their expectation about service recovery to complete the service recovery process.” When customers are engaged in the service recovery process, it will give them the feeling of honor and they give more feedback to webstore for the improvement of their service. Customer’s assistive intent not only helps webstore to make efficient service recovery strategies during COVID situation. It will also help webstore to get loyal customers in post-COVID economic situation.

## Limitations and Future Recommendations

The current study was conducted during the pandemic. Therefore, most of the respondents were the students at a university. Different age groups and people from different occupations might help the future research to explore more recovery strategies. We used cross-sectional data for this longitudinal research in different time spans, which might give better results. Further, this research was conducted in Malaysia and our respondents were Malaysian customers. Future studies might be conducted by taking more respondents from different countries, and a comparative study might be helping to understand the consumer behavior regarding online shopping during COVID situation. Future studies also can include different online channels to conduct the study. By developing service failure and service recovery scenarios, the findings can be checked empirically. Based on our finding, we found that customer’s assistive intent might play a positive role in retaining angry customers. Customers’ assistive intent is a new variable that has not been tested in service recovery context. By scale development of customer’s assistive intent, researchers can get better results to improve service recovery strategies.

## Data Availability Statement

The original contributions presented in the study are included in the article/supplementary material, further inquiries can be directed to the corresponding author/s.

## Ethics Statement

Ethical review and approval was not required for the study on human participants in accordance with the local legislation and institutional requirements. The patients/participants provided their written informed consent to participate in this study.

## Author Contributions

MM contributed to conception or design of the work and drafting the article. UT and DT contributed to data collection. MA and MM contributed to data analysis and interpretation. AH and MA facilitated critical revision of the article. AH and MM helped in proofreading to improve the quality of the manuscript. All authors contributed to the article and approved the submitted version.

## Conflict of Interest

The authors declare that the research was conducted in the absence of any commercial or financial relationships that could be construed as a potential conflict of interest.

## Publisher’s Note

All claims expressed in this article are solely those of the authors and do not necessarily represent those of their affiliated organizations, or those of the publisher, the editors and the reviewers. Any product that may be evaluated in this article, or claim that may be made by its manufacturer, is not guaranteed or endorsed by the publisher.
